# Acute Moderate Exercise Does Not Further Alter the Autonomic Nervous System Activity in Patients with Sickle Cell Anemia

**DOI:** 10.1371/journal.pone.0095563

**Published:** 2014-04-16

**Authors:** Mona Hedreville, Keyne Charlot, Xavier Waltz, Stéphane Sinnapah, Nathalie Lemonne, Maryse Etienne-Julan, Valérie Soter, Olivier Hue, Marie-Dominique Hardy-Dessources, Jean-Claude Barthélémy, Philippe Connes

**Affiliations:** 1 Laboratory ACTES (EA 3596), Department of Physiology, French West Indies and Guiana University, Pointe-à-Pitre, Guadeloupe, France; 2 Emergency Care Department, Academic Hospital of Pointe-à-Pitre, Pointe-à-Pitre, Guadeloupe, France; 3 UMR Inserm U1134 French West Indies and Guiana University, Pointe-à-Pitre, Guadeloupe, France; 4 Laboratoire d’Excellence du Globule Rouge (LABEX GR-Ex), PRES Sorbonne, Paris, France; 5 Sickle cell Center, Academic Hospital of Pointe-à-Pitre, Pointe-à-Pitre, Guadeloupe, France; 6 Direction of Research and Innovation, Academic Hospital of Pointe-à-Pitre, Pointe-à-Pitre, Guadeloupe, France; 7 Laboratory EA4607 SNA-EPIS, Jean Monnet University of Saint-Etienne, PRES Lyon, Saint-Etienne, France; 8 Institut Universitaire de France (IUF), Paris, France; Temple University, United States of America

## Abstract

A decreased global autonomic nervous system (ANS) activity and increased sympathetic activation in patients with sickle cell anemia (SCA) seem to worsen the clinical severity and could play a role in the pathophysiology of the disease, notably by triggering vaso-occlusive crises. Because exercise challenges the ANS activity in the general population, we sought to determine whether a short (<15 min) and progressive moderate exercise session conducted until the first ventilatory threshold had an effect on the ANS activity of a group of SCA patients and a group of healthy individuals (CONT group). Temporal and spectral analyses of the nocturnal heart rate variability were performed before and on the 3 nights following the exercise session. Standard deviation of all normal RR intervals (SDNN), total power, low frequencies (LF) and high frequencies powers (HF) were lower but LF/HF was higher in SCA patients than in the CONT group. Moderate exercise did not modify ANS activity in both groups. In addition, no adverse clinical events occurred during the entire protocol. These results imply that this kind of short and moderate exercise is not detrimental for SCA patients.

## Introduction

Sickle cell anemia (SCA) patients are marked by several biological abnormalities such as decreased red blood cells (RBC) deformability [1]–[5], increased RBC aggregates strength [2], [3], [6] and inflammation [7], and a pro-oxidant state [8], which play a role in several complications. However, they are also characterized by an alteration of the autonomic nervous system (ANS) activity (i.e., parasympathetic withdrawal and sympathetic predominance), determined by the analysis of heart rate variability (HRV) in resting condition [9], [10], with the degree of alteration reflecting the clinical severity [11]–[13]. Nebor et al [11] demonstrated that SCA patients at high risk for developing painful vaso-occlusive crises have a large parasympathetic activity withdrawal in comparison with less severe SCA patients. This sympatho-vagal imbalance may further exacerbate vaso-occlusive crises by increasing peripheral vasoconstriction [10].

While regular physical activity has been proven to be a clinical strategy able to decrease co-morbidity and provide health benefits in several chronic diseases such as obesity, diabetes or asthma [14]–[16] health care professionals have not yet considered a clinical benefit of physical exercise for patients with SCA [17]. Depending on its duration and intensity, exercise may promote lactic acidosis, tissue hypoxia and/or dehydration, which are conditions known to stimulate the polymerization of the abnormal hemoglobin (HbS) and RBC sickling, hence triggering vaso-occlusive events [18]. Nevertheless, few studies demonstrated that exercises lasting less than 30 min at intensity lower than 75% of predicted maximal heart rate (HR) were well tolerated by SCA patients regarding hemorheological, inflammatory, vascular and oxidative stress parameters [19]–[21]. Moreover, we recently demonstrated that a short (less than 15 min) exercise conducted until the first ventilatory threshold (VT1; moderate intensity) did not cause supplemental alterations in RBC deformability, blood viscosity or coagulation markers in SCA patients [1]. In healthy individuals, exercise is known to challenge the ANS activity with a wide parasympathetic withdrawal during, but also several hours after the effort [22]. Usually, a rebound of the parasympathetic activity is observed two days after an exercise bout, which may confer cardiovascular protection [22]. There is an increasing need to define, in terms of intensity and duration, what kind of exercise SCA patients may perform without any risk for developing acute complications. Thus, the aim of this study was to assess the effects of a short (<15min) and moderate exercise (progressive session conducted until the first ventilatory threshold) on the ANS activity of a group of SCA patients and a group of healthy individuals. Incremental exercise is widely used in several chronic diseases to screen for cardiorespiratory and/or peripheral disorders, as well as to define intensity for training programs, but has been rarely utilized in SCA, and it is unknown whether this classical exercise is safe or not for this population. The demonstration that an incremental exercise conducted to the first ventilatory threshold would be safe for SCA patients could, then, be used to propose individualized exercise rehabilitation program in the near future.

## Materials and Methods

### Subjects

Seven patients with SCA (4 males and 3 females, age: 33.3±10.8 yrs, weight: 63.0±12.7 kg, height: 173±7 cm, hematocrit: 22.7±4.3%, white blood cells: 9.37±2.13 10^9^/l, RBCs: 2.80±0.53 10^12^/l, hemoglobin concentration: 1.33±0.44 g/dl) and a control group of 9 subjects with normal hemoglobin (CONT; 5 males and 4 females, age: 34.8±8.4 yrs, weight: 75.8±11.8 kg and height: 172±8 cm) agreed to participate in the present study. All the SCA patients recruited were regularly followed by the Sickle Cell Unit of the Academic Hospital of Pointe-à-Pitre (Guadeloupe) and were in steady-state condition at the time of the study. Patients considered to be severe (*i.e.*, either >3 hospitalizations per year for vaso-occlusive crisis, >1 acute chest syndrome or >1 transfusion per year during the last 5 years) were excluded. Patients with other hemoglobinopathies than SCA, cardiovascular disorders, positive history of stroke, pulmonary hypertension, diabetes, body mass index (BMI) ≥30 kg/m^2^, under hydroxyurea therapy, smokers and/or pregnant women were also excluded. Before enrollment, all patients and control subjects had clinical examination with anthropometric measurements and underwent resting electrocardiography, echocardiography and blood pressure measurements to check for the absence of severe exercise contraindication. All participants received verbal and written explanation of the objectives and procedures of the study and subsequently provided written informed consent. The study was approved by the Regional Ethics Committee (CPP Sud-Ouest Outre-Mer III, Bordeaux, France; Registration number: SNAD - 2010-A00126-33). The experiments were performed in accordance with the guidelines set by the Declaration of Helsinki.

### Protocol

Each SCA patient and CONT subject performed an incremental exercise until VT1, as previously described [1]. HRV was measured overnight, the night preceding the exercise session (D-1) and during the 3 following nights after the effort (D0, D1 and D2). Subjects were asked to refrain from alcohol and caffeine consumption and, although not evaluated by specific physical activity questionnaires, from physical exercise from the 3 days before D-1 and until the end of the experiment.

### Acute Exercise

The exercise protocol has been previously described in details [1]. Briefly, the cycling exercise (Welch Allyn cycloergometer, USA) test, conducted between 1∶00 and 3∶00 PM, consisted of 3 minutes warm-up at 10 W for SCA and 20 W for CONT, and then, the load was increased every minute (*i.e*., SCA: 5–7 W; CONT: 15–30 W) until VT1 was reached. The power increment used for SCA patients was close to the one previously used by Callahan et al. [23]. Blood pressure measurements, heart rate and pulse oxymetry monitoring were strictly performed by an experienced cardiologist in the line of the recommendations of the French Society of Cardiology. Gas exchanges were measured with a breath-by-breath automated exercise metabolic system (Oxycon Mobile, Jaeger, Germany) to determine the appearance of VT1 during exercise (see [1]). Pedaling speed remained constant at 60–70 rotation per minute during the exercise test.

### Autonomic Nervous System Activity

During the 4 nights of measurement, a holter electrocardiograph (Novacor system, Duosoft, France) was placed on the chest of each patient after clinical examination and recording of the inter-beat (RR) intervals was performed from 6∶00 p.m. to 8∶00 a.m. RR intervals were visually inspected, corrected if presence of artifacts, then validated before analysis. Only the night periods were analyzed (midnight to 7 a.m.) to avoid variations arising from differences in the subject’s daily environment. HRV recordings during the nights avoid variations due to daily activities, which may vary from one day to another, which would decrease the signal (variations caused by the exercise) to noise (variations due to the daily activities) ratio [24]. The large period of recordings analyzed covers several full sleep cycles including thus a similar repartition of sleep states between subjects. This allows comparison between successive nights and subjects. Artifacts were replaced by the mean of the two neighbors RR. Resulting RR signals are then re-sampled at 2 Hz using a cubic spline interpolation. A temporal analysis of HRV was performed to calculate the standard deviation of all normal RR intervals (SDNN) and the square root of the mean squared differences between adjacent normal RR intervals (RMSSD). After fast Fourier transform (FFT), the power spectrum indices were calculated as recommended by the Task Force of the European Society of Cardiology and the North American Society of Pacing and Electrophysiology [25]. The FFT is based on the fact that data present with a stationary organization and are a combination of sinusoidal functions [26], [27]. Thus algorithms of analysis are searching for sinusoidal similarities in the signal. The search does not indicate the localization of the particular frequency along the observed signal, but, instead, provides a cumulate spectrum power of a particular frequency, i.e., corresponding to the number of occurrences of the given sinusoidal function. The low frequencies (LF, 0.04–0.15 Hz) are known to reflect both sympathetic and parasympathetic activities. The high frequencies (HF, 0.15–0.40 Hz) reflect parasympathetic activity. The LF/HF ratio, which was used as a broad index of “sympathovagal balance” [25], and the total frequency power (Ptot) were also calculated. SDNN and Ptot reflected the global autonomic activity, the LF/HF ratio has been proposed as a marker of ANS balance and the RMSSD is strongly related to HF power [28], [29].

### Statistics

The results are presented as mean ± standard deviation (SD). The time courses of temporal and spectral indices of HRV were compared between the two groups using a two-way analysis of variance (ANOVA) with repeated measures after log transformation of the data. However, results are presented in absolute value. Pair-wise contrasts were used when necessary to locate where significant differences occurred. The significance level was defined as p<0.05. Analyses were conducted using Statistica (v. 5.5, Statsoft, Tulsa, OK, USA).

## Results

**Figure 1:**
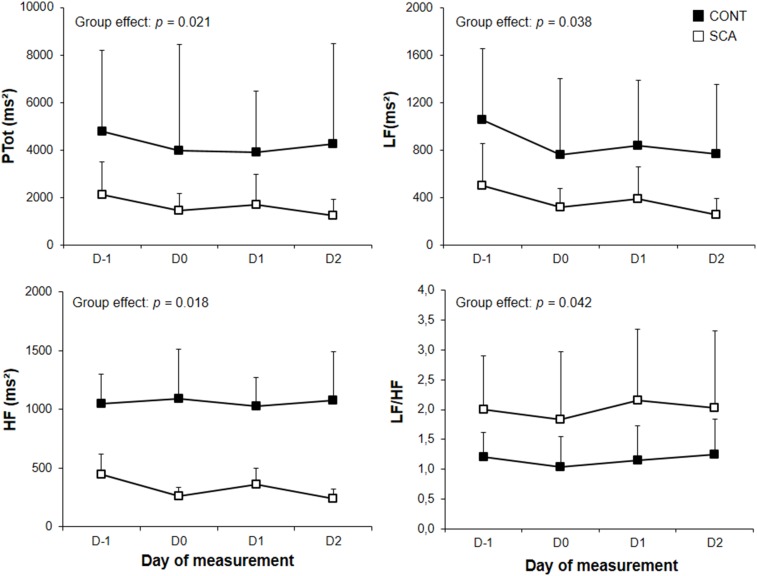
Spectral indices of heart rate variability. D-1: the night before exercise, D0: the night just after exercise, D1 and D2: the nights 1 and 2 days after exercise. Ptot: Total Power, LF: low frequencies, HF: high frequencies.

Statistical analysis revealed that SDNN (group effect: p = 0.048), RMSSD (group effect: p = 0.048), Ptot (group effect: p = 0.05021), LF (group effect: p = 0.037) and HF (group effect: p = 0.05018) levels were lower and LF/HF levels (group effect: p = 0.042) higher in the SCA group than in the CONT group (Table 1; Figure 1). Neither a time effect, nor a time x group interaction was found for all indices (Table 1; Figure 1). Although oxygen uptake, ventilation and power were lower in SCA patients than CONT subjects at VT1 (data not shown, see [1]), HR (133±21 vs 136±19 bpm for CONT and SCA, respectively) and mean arterial pressure (101.2±13.2 vs 100.6±15.6 mmHg for CONT and SCA, respectively) were similar. Except one SCA individual who exhibited a 11% decrease of hemoglobin oxygen saturation compared to baseline, the other individuals (CONT and SCA) did not exhibit significant (i.e., more than 4%; [1]) change. Using the Borg scale [30] of perceived exertion, SCA patients and controls reported that the feeling of effort, strain, discomfort and/or fatigue was mild to moderate (score ranging from 11 to 13).

**Table 1:** Temporal indices of heart rate variability.

		D-1	D0	D1	D2
HR (bpm)	CONT	62.4±10.9	62.2±9.8	61.3±9.7	65.0±16.4
	SCA	65.6±5.1	67.8±3.8	69.3±7.0	71.7±5.4
SDNN (ms)	CONT	121±39	106±48	119±33	114±41
	SCA*	96±28	80±19	87±24	76±18
RMSSD (ms)	CONT	74±30	70±44	71±31	69±46
	SCA*	46±29	39±18	41±24	36±20

Values are mean ± SD. D-1: the night before exercise, D0: the following night after exercise, D1 and D2: the nights 1 and 2 days after exercise, HR: Heart rate, SDNN: standard deviation of all normal RR intervals, RMSSD: square root of the mean squared differences between adjacent normal RR intervals. *Significant group effect (*p<*0.05).

## Discussion

The aim of the present study was to test the effect of a single moderate (progressive session conducted until the first ventilatory threshold) and short exercise (<15 min) on the ANS activity of SCA patients. The physiological responses indicate that the exercise represented the same relative physiological stress for SCA patients and CONT subjects despite different aerobic capacities. Although two patients of the SCA group exhibited paroxystic and non-severe arrhythmia, the exercise was well tolerated.

Baseline ANS activity has been reported to be altered in SCA patients compared to healthy subjects [9]–[11], which is in agreement with our findings since all HRV parameters were lower in the SCA group than in the CONT group. Moreover LF/HF levels were higher in the SCA group indicating that the sympathetic activity was predominant due to a parasympathetic withdrawal. The reasons of these differences are not yet fully understood [5]. However, it has been suggested that chronic anemia may lead to a persistent sustained decrease in autonomic fluctuations [29]. Moreover, repeated hypoxemic episodes, as it can be the case in SCA patients [31], may increase sympathetic activity and decrease parasympathetic activity [32]. Very short and transient hypoxic stress stimulus has been reported to induce parasympathetic withdrawal in SCA patients but not in healthy individuals [33]. The role of hypoxemia/hypoxia on ANS dysfunction has been reinforced by studies in rats showing that chronic hypoxia causes cell loss in the nucleus ambiguous, a structure from which several vagal efferent axons innervate ganglionated plexuses in the dorsal surface of cardiac atria, which in turn may have different functional roles in cardiac regulation [34]. SCA patients with the most severe form of the disease [10], at risks for developing frequent vaso-occlusive crises [11] or acute chest syndrome [12], or with leg ulcers and erectile dysfunction [35], have a depressed ANS activity and a reduced parasympathetic activity. These findings suggest a role of parasympathetic withdrawal and sympathetic predominance in the pathophysiology of several sickle cell complications. For example, altered autonomic tone has been suspected to exacerbate pain episodes in SCA patients by increasing peripheral vasoconstriction [10].

Whether moderate exercise increases the predominance of the sympathetic activity over the parasympathetic activity is of great concern for SCA patients. In healthy individuals, a single intense exercise session usually alters ANS activity within the first night following exercise [22], [36]; HF being lower than before the effort. Then, on the second night after the effort, some authors noted a return to baseline of the ANS activity [36] or even a positive rebound of the parasympathetic activity conferring greater cardiac and vascular protection than before exercise [22]. Myllymäki et al. [37] showed, in healthy individuals, that a 90-min exercise session at moderate intensity (60% VO_2max_) decreased ANS activity on the night following exercise while a 30-min session at high (75% VO_2max_), moderate or low intensity (45% VO_2max_) or a 60-min session at moderate intensity had no impact. Indeed, in healthy subjects, exercise duration seems to be more important than intensity to really challenge ANS on the night following exercise, and the absence of ANS activity fluctuations after exercise in the CONT group was probably related to the too short exercise duration (<15 min) [37]. In SCA patients, prolonged and intense exercise bouts are usually dangerous since they may trigger red blood cell sickling and painful vaso-occlusive crises [17]. The lack of change in the ANS activity of SCA patients, which are not involved in any physical activity, after the exercise suggests that the effort proposed 1) is safe and well tolerated by this population (no adverse clinical event occurred during the entire protocol), with no further alterations in ANS activity but, 2) is probably not intense and/or prolonged enough to promote positive autonomic adaptations as it can be the case, sometimes, for healthy subjects [22].

In conclusion, this study shows that a short (<15 min duration) single exercise session performed until the first ventilatory threshold has no positive impact on ANS activity but most importantly did not further alter the ANS activity of SCA patients with a mild clinical expression of the disease. These results, with those of Waltz et al. [1], strongly suggest that this kind of exercise could be safely performed in SCA patients with. While we analyzed the ANS activity several hours and days after the exercise bout, we did not investigate the immediate effects of exercise on ANS activity in SCA patients. Our methodological choice was motivated by the findings of Furlan et al. [38] who demonstrated a decrease of ANS activity the night following heavy exercise, with this condition representing a vulnerability status [39]. However, Jouven et al. [40] also demonstrated that the time for heart rate to recover immediately after exercise was a predictor of sudden death in the general population. Indeed, it might be of interest to accurately analyze the heart rate kinetics during and immediately after exercise in SCA patients. Based on the marked parasympathetic withdrawal observed in SCA patients, one could expect a slow heart rate recovery following exercise in this population. Future studies should also investigate whether chronic light exercise (i.e., exercise rehabilitation program) could have beneficial effects on ANS activity in SCA patients with various degrees of clinical severity. It could encourage health care professionals to prescribe individualized and regular physical activity as a treatment strategy in SCA, as it is already the case in other chronic diseases like type 2 diabetes [14], asthma [15], chronic obstructive pulmonary disease or cardiac heart failure [16]. Finally, further studies are needed to examine sex differences in autonomic responses to exercise in SCA patients. The effect of menstrual cycle and hormones variations on the ANS activity is very controversial [41], [42]. Nevertheless, one study reported an association between estrogen level and several parameters reflecting ANS activity, even if the level of parasympathetic and sympathetic activities did not change between the different phases of the menstrual cycle [42]. In another study, the sympathetic activity, measured by muscle sympathetic nerve activity, changed with the menstrual activity and the level of estrogen, but this association was blunted during handgrip exercise [43]. Indeed, although the data available in the literature regarding the role of sex hormones and menstrual cycle on ANS activity in response to exercise are sparse and conflicting, we believe there is a need to address this issue in women with SCA.
